# Sex Differences in Youth with Chronic Tic Disorder and Tourette Syndrome: Evaluation of Tic Severity, Psychological Profiles, and Quality of Life

**DOI:** 10.3390/jcm13092477

**Published:** 2024-04-24

**Authors:** Méliza Gagnon, Ilana Singer, Simon Morand-Beaulieu, Kieron P. O’Connor, Bruno Gauthier, Douglas W. Woods, Pierre Blanchet, Marc E. Lavoie, Julie B. Leclerc

**Affiliations:** 1 Department of Psychology, Université du Québec à Montréal, Montreal, QC H2X 3P2, Canada; singer.ilana@courrier.uqam.ca (I.S.); morand-beaulieu.simon@courrier.uqam.ca (S.M.-B.); leclerc.julie@uqam.ca (J.B.L.); 2 Centre de Recherche du Centre Intégré Universitaire de Santé et de Services Sociaux (CIUSSS) du Nord-de-l’Île-de-Montréal, Montreal, QC H1E 1A4, Canada; 3 Centre de Recherche de l’Institut Universitaire en Santé Mentale de Montréal, Montreal, QC H1N 3V2, Canadapierre.j.blanchet@umontreal.ca (P.B.); marc.lavoie@teluq.ca (M.E.L.); 4 Department of Psychology, Université de Montréal, Montreal, QC H2V 2S9, Canada; bruno.gauthier@umontreal.ca; 5 Department of Psychology, Marquette University, Milwaukee, WI 53233, USA; douglas.woods@marquette.edu; 6 Department of Stomatology, Université de Montréal, Montreal, QC H3T 1J4, Canada; 7 Department of Social Sciences, Literature and Communication, Université TÉLUQ, Montreal, QC G1K 9H6, Canada

**Keywords:** sex differences, youth, chronic tic disorder, Tourette syndrome, tic onset, tic severity, action planning styles, quality of life, externalizing symptoms, internalizing symptoms

## Abstract

**Background**: Tourette syndrome (TS) and Chronic Tic Disorder (CT) are neurodevelopmental conditions involving motor and/or phonic tics. Youth with tics may encounter feelings of isolation, diminished self-esteem and quality of life, and academic difficulties. A growing body of scientific literature suggests sex differences in youth with tics, but findings have been mixed so far. Because symptom severity peaks around puberty, understanding sex differences in tic manifestations and associated symptoms during this critical period is essential. Therefore, we aimed to assess sex differences related to tic symptoms, action planning styles, quality of life, and externalizing/internalizing symptoms in youth with tics. **Methods**: Our sample consisted of 66 youths with tics (19 girls) aged 7–14 (mean = 10 years). Youths were assessed with clinical interviews, as well as self- and parent-reported inventories evaluating tic symptoms, psychological profiles, and quality of life. **Results**: While no differences in tic symptoms were found, girls exhibited lower functional inflexibility, reduced overall functional planning effectiveness, and higher impairment in the psychological well-being subscale than boys. Additionally, girls had reduced general life satisfaction and social self-esteem. Boys reported more explosive outbursts, higher levels of hyperactivity, and more difficulties with self-concept. **Conclusions**: Our analyses suggested differences in several manifestations associated with tics. This introduces new perspectives that refine our understanding of sex differences. A better understanding of sex differences in tic disorders may eventually improve outcomes for all individuals living with these conditions.

## 1. Introduction

Chronic Tic Disorder (CT) and Tourette syndrome (TS) are neurodevelopmental conditions classified as persistent primary tic disorders [1]. CT involves the presence of either motor or phonic tics, whereas TS is characterized by several motor tics and at least one phonic tic. In both diagnoses, tics persist for at least a year, with the onset occurring before age 18 [1]. Over time, tics change in frequency, intensity, and location, with important variations across individuals [1]. Tics can manifest in various forms, ranging from simple actions, such as eye blinking or throat clearing, to more complex behaviors, such as the involuntary imitation of movements or repeating words. Tic onset occurs most often between the ages of 4 and 6, and a peak in tic severity is typically seen around age 11 [1,2]. The prevalence rates of CT and TS are respectively around 1.6% and 0.8% in youths, with boys being up to four times more affected than girls [3,4,5]. This emphasizes the importance of considering sex differences in understanding the diverse clinical presentations of tic disorders. 

The intricate nature of these conditions extends beyond the tics themselves, encompassing a range of cognitive, behavioral, and psychiatric challenges. Action planning styles, characterized by an excessive preparatory mental organization and anticipation, inflexibility in adapting to changes, and a tendency towards overactivity leading to inefficiency in task prioritization and execution, may lead to an increase in tics under certain conditions [6,7]. This idea stems from O’Connor’s cognitive-behavioral model, which states over-preparedness, which refers to excessive mental organization and scenario planning, as well as over-anticipation, suggesting a constant mental state of readiness for potential future problems, can both exacerbate and be exacerbated by tics [6,7]. This may also occur through inflexibility, manifesting as a rigid adherence to plans or routines that makes adapting to unforeseen changes challenging. Simultaneously, overactivity may cause individuals to start multiple tasks without clear prioritization, leading to a dispersed focus and inefficiency. In addition to tics, 20% to 67% of individuals with tic disorders experience explosive outbursts [8]. These manifestations are characterized by a sudden onset of physical or verbal aggression, disproportionate to the triggering stimulus, and a feeling of loss of control [9]. Moreover, up to 90% of people with TS present with psychiatric comorbidities, such as attention-deficit hyperactivity disorder (ADHD), obsessive-compulsive disorder (OCD), anxiety disorders, and mood disorders [10,11,12]. As a result, youth affected by these disorders may face challenges that contribute to feelings of isolation, diminished self-esteem, academic or vocational difficulties, and an overall reduction in their quality of life [10,13,14]. The complexity of managing tic disorders is further amplified by potential sex differences, introducing additional layers of variability in the clinical presentation of youth with tics. 

The existing scientific literature on differences in tic manifestations between sexes in youth shows considerable variability. Some studies suggest that boys exhibit an earlier onset of tics than girls [15,16], while others find no significant differences [17,18]. Furthermore, studies indicate variation in the manifestation of motor and phonic tics between boys and girls, with patterns and severity differing in ways that are specific to each sex. Some research indicates a greater severity of motor tics in boys than girls [17,18,19]. However, other studies observe the opposite, suggesting that girls may exhibit a higher severity of motor tics than boys [20,21]. In the realm of phonic tics, research findings diverge further. Two studies report a higher prevalence or severity of phonic tics in boys [17,18], while another suggests that girls might have more specific phonic tics, such as sniffing or grunting [21]. Meanwhile, another study reports no significant sex differences in phonic tics [19]. Similarly, the assessment of global tic severity reveals inconsistencies across studies, with some reporting higher global tic severity in boys [18,19], while others find it more pronounced in girls [20,21], and yet another study reports no significant sex differences [22]. An interaction between sex and age in youth with TS appears to impact tic severity, but further studies are required for a more comprehensive understanding [19,23]. Studies on tic-related impairment also yield conflicting results, as one suggests higher tic-related impairment among girls [21], and another indicates comparable levels of tic-related impairment between boys and girls [24]. The substantial variability in participant characteristics may contribute to the disparities in current research findings. Moreover, the inconsistency in the use of measurement tools across studies further complicates the interpretation of these results. Addressing the methodology and participant selection criteria in these studies is crucial for a deeper understanding of the results and their implications.

Studies focusing on externalizing and internalizing symptoms associated with tic disorders in youth also present conflicting findings regarding sex differences. For instance, it is unclear whether boys are more prone to explosive outbursts than girls [25,26]. However, there is a consensus among studies that boys with TS tend to manifest more symptoms of disruptive behavior compared to girls [19,23]. Furthermore, a study reports that girls under the age of 12 exhibit more externalizing symptoms than boys, but these symptoms seem to become similar between sexes in adolescence [27]. Most studies indicate that boys with TS appear to present more ADHD symptoms compared to girls [17,18,19,20,23], although one study reported similar prevalences of ADHD diagnoses in both sexes [27]. Associated internalizing symptoms appear to be more common in girls with TS, as some studies reported more emotional problems and anxiety and mood disorders [19,20,23,27], although others report similar internalizing problems in both sexes [20,27]. OCD symptom severity appears to be similar between girls and boys with TS [17,18,19,20,27]. 

In light of the considerable variability and conflicting findings reported in existing literature regarding sex differences in tic manifestations among youth, there is a clear need for further investigation. Challenges such as the underrepresentation of girls in research samples and the inconsistency in clinical presentations highlight gaps in our current understanding. Additionally, to our knowledge, no study has specifically investigated sex differences in action planning styles among youth with tic disorders, nor has there been an examination of the differential impact of tics on the quality of life of boys and girls. These are important aspects to consider, given their potential to significantly influence the clinical profiles and therapeutic approaches for youth with these conditions. Therefore, the purpose of the present study was to build upon prior findings and offer new insights into observed contradictions, focusing specifically on a treatment-seeking sample. The specific aims of this study were to assess sex differences, as well as interactions between age and sex regarding tic severity, action planning styles, quality of life, and internalizing and externalizing symptoms in youth with persistent tic disorders. Enhancing our understanding of sex differences in tic disorders is crucial, as it may have implications for diagnostic criteria, treatment effectiveness, and understanding lifetime trajectories of these disorders. Because tic severity typically peaks around age 11, gaining a more comprehensive understanding of tic manifestations before, during, and after this critical period is essential.

## 2. Materials and Methods

### 2.1. Participants

The participants in the study were children and adolescents enrolled in a randomized controlled trial (RCT) comparing the efficacy of two behavioral therapies for tics [28]. Inclusion criteria consisted of (i) aged 7 to 14 years old and (ii) having simple or complex tics persisting for at least one year and occurring daily, encompassing both CT and TS diagnoses. Notably, the inclusion criteria allowed for the participation of individuals who were receiving medication for their tics, provided that their medication dosage had remained stable for a minimum of three months prior to their enrollment in the study. Exclusion criteria consisted of (i) a history of head injury resulting in sensory-motor impairment, (ii) a diagnosis of autism spectrum disorder or intellectual disability (IQ < 75), (iii) personality disorders, (iv) neurological disorders such as Parkinson’s disease or hemifacial spasms, (v) alcohol/substance abuse, and (vi) current treatment for tics (other than medication). Comorbidities, such as ADHD, OCD, anxiety, and depression, that did not necessitate current treatment were not grounds for exclusion. All participants read and signed informed consent, and the local institutional ethics board approved the project (project #MP-12-2016-262).

### 2.2. Procedures

The current study utilized data collected from assessments of both youth participants and their parents. Prior to their participation, the procedures of the RCT were explained, ensuring informed consent. Evaluators, who received extensive training in administrating semi-structured interviews and clinical global assessments, conducted evaluations at baseline. To maintain the integrity and confidentiality of the study, evaluators signed agreements prohibiting the discussion of case specifics with uninvolved team members. 

The evaluation began with a telephone screening for suitability, followed by a comprehensive face-to-face clinical assessment, lasting approximately three hours. Diagnoses were made by independent specialists prior to the study. Youth diagnosed with CT presented either motor or phonic tics daily for at least one year. Youth diagnosed with TS were accurately diagnosed, with TS being the primary presenting issue. Additionally, participants filled out paper form questionnaire measures at the *Institut universitaire en santé mentale* research center in Montreal, a process that took 1 to 2 h. Participants were compensated for attendance.

### 2.3. Clinical Assessment

The Yale Global Tic Severity Scale (YGTSS) [29] is a clinician-rated scale used to evaluate the severity of motor and phonic tics over the last week. Clinicians interviewed both children and their parents separately via a structured interview. Tics were assessed across five dimensions, including number, frequency, intensity, complexity, and interference on a scale of 0 (none) to 5 (always). The scale generates individual scores for both motor and vocal tics, ranging up to 25 each. The addition of the latter two subscales results in the total tic score (range: 0–50). There is also an impairment scale (range: 0–50). The global tic score (range: 0–100) is obtained by adding the total tic score and the impairment score.

### 2.4. Questionnaire Measures

A sociodemographic and clinical questionnaire was used to collect primary data on participants. From this questionnaire, essential information was gathered, focusing on key details such as age, sex, age of tic onset, and presence of explosive outbursts (yes or no). 

The Style of Planning Questionnaire (STOP) [6] was used to assess action planning styles. This questionnaire highlights behavioral and cognitive strategies and uses a novel approach by offering choices between overactive and non-overactive responses to planning scenarios (e.g., choosing to start multiple tasks simultaneously or pausing to prioritize). It features four scales, including over-preparedness (range: 0–50); inflexibility (range: 0–70); over-anticipation (range: 0–40); and overactivity (range: 0–10). Additionally, there is a global scale to assess overall planning effectiveness (range: 0–180). The STOP includes 18 items and asks participants to rate their preferred planning style on a 10-point scale, distinguishing between dysfunctional (0 to 4.99) and functional (5.01 to 10) approaches, with a score of 5 indicating no preference between the two styles. 

The Gilles de la Tourette Syndrome-Quality of Life Scale (GTS-QoL) [14,30] was used to assess the impact of tics on quality of life among youth with CT and TS. The GTS-QoL is a 27-item inventory assessing quality of life across psychological, physical, cognitive, and obsessive-compulsive subscales. Subscale scores are transformed to a range of 0 to 100. Each item is assessed on a 5-point Likert scale ranging from 0 (never) to 4 (always). At the end of the questionnaire, an additional question assesses life satisfaction (range: 0–100).

The Beck Youth Inventories, 2nd edition (BYI-II) [31], was used to evaluate psychological symptoms in youth, specifically focusing on both internalizing experiences, such as self-concept, anxiety, and depression, as well as externalizing experiences, like anger and disruptive behaviors. Each inventory consists of 20 questions about thoughts, feelings, and behaviors that are related to emotional and social impairment in the last two weeks (range: 0–60). The BYI-II has Likert-type questions ranging from 0 (never) to 3 (always). The higher the score, the greater the impairment. 

The Culture-Free Self-Esteem Inventories, 2nd edition (CFSEI-II) [32], was used to evaluate self-esteem, an internal experience frequently affected in the context of tic disorders. The CFSEI-II is a measure designed to assess self-esteem in youth across four distinct domains, including general self-esteem (range: 0–10); parental or at-home self-esteem (range: 0–5); social self-esteem (range: 0–5); and academic self-esteem (range: 0–5). In addition to these domain-specific scores, the inventory includes a global score (range: 0–30), which provides an overall measure of self-esteem. The CFSEI-II also includes lie items (range: 0–3), which are designed to detect and control for response bias, ensuring that the responses are genuine and reflective of their true feelings and perceptions. For each item, they were asked to indicate whether the question corresponds to their situation (yes or no).

The Conners’ Parent Rating Scale—Revised: Short Version (CPRS-R:S) [33] was used to evaluate ADHD symptoms. The CPRS-R:S consists of 27 questions related to school, family, and social background. It provides a comprehensive and reliable assessment of ADHD according to the Diagnostic and Statistical Manual of Mental Disorders (5th ed.; DSM-5) [34] diagnostic criteria. The CPRS-R:S is divided into three scales, including oppositional and hyperactivity, which assess external behaviors observable in various contexts, and cognitive problems/inattention, which focuses on internal symptoms reflecting the child’s attentional challenges (range: 0–18). Additionally, it includes an ADHD index (range: 0–36), which allows for the identification of youth at risk for ADHD. Parents of children with CT and TS completed the CPRS-R:S and were asked to quantify their child’s behavior on a 4-point Likert scale ranging from 0 (not at all true) to 3 (very true). 

### 2.5. Statistical Analyses

Statistical analyses were conducted with SPSS version 27. Categorical data were tested using chi-square tests comparing boys and girls. The normality assumption of continuous data was assessed using the Shapiro–Wilk test. *t*-tests were used to compare variables across sexes for normally distributed continuous variables. Mann–Whitney U tests were used instead for non-normally distributed continuous outcome variables. Correlations between age and our variables of interests within each respective sex were measured using Pearson or Spearman correlation coefficients, depending on the normality of the data. One-way ANOVAs were used to assess the interactions between age and sex. The significance level for all analyses was set at α = 0.05.

## 3. Results

### 3.1. Tic-Related Measures

The current study sample comprised 66 participants (19 girls) aged 7–14 (mean = 10 years). Regarding medication intake, 43.08% of the sample reported using stable medication, as described in the inclusion criteria. More precisely, 10.77% were prescribed antipsychotics (aripiprazole, risperidone), 20.00% were taking alpha-agonists (clonidine, guanfacine extended-release), 27.69% were using ADHD medication (stimulants, atomoxetine), 4.62% were on SSRIs (fluoxetine), 1.54% were prescribed benzodiazepine (clonazepam), and 1.54% were using antibiotics (amoxicillin). Table 1 presents the means and standard deviations (SD) of age at tic onset, clinical assessment of tic severity, action planning styles, and quality of life related to tics. The analysis revealed no significant difference in age at tic onset between girls and boys. Similarly, assessments of motor tic severity, phonic tic severity, total tic severity, impairment rating, and global severity conducted with both youth and their parents showed no significant differences between girls and boys.

Regarding action planning styles, girls exhibited significantly lower functional inflexibility levels than boys (39.8 ± 8.3 vs. 44.1 ± 6.2; t (62) = −2.12, *p* = 0.038). Additionally, girls demonstrated significantly lower overall functional daily planning effectiveness than boys (102 ± 14.3 vs. 114 ± 16.9; U = 255, *p* = 0.017). However, no significant differences between girls and boys regarding over-preparedness, anticipation, and over-activity approaches were found.

Regarding the impact of tics on quality of life, girls experienced significantly higher impairment in the psychological well-being subscale compared to boys (39.4 ± 20.4 vs. 26.6 ± 14.6; U = 252, *p* = 0.015). Additionally, girls had significantly greater impairment in general life satisfaction than boys (31.6 ± 14.7 vs. 24.0 ± 12.1; t (62) = 2.12, *p* = 0.038). No significant differences were found between girls and boys in physical, obsessive-compulsive, and cognitive subscales.

### 3.2. Externalizing Symptoms

Table 2 presents the means and standard deviations (SDs) of externalizing symptoms. A significantly greater number of boys reported experiencing explosive outbursts compared to girls (56.5% vs. 26.3%; χ^2^ = 4.92, *p* = 0.027). No significant differences were found between girls and boys in the anger and disruptive behavior subscales. Boys exhibited significantly higher levels of hyperactivity compared to girls (5.8 ± 3.9 vs. 3.7 ± 3.2; U = 252, *p* = 0.030). No significant differences were found between girls and boys in oppositional behaviors or in the ADHD index. 

### 3.3. Internalizing Symptoms

Table 3 presents the means and standard deviations (SDs) of internalizing symptoms. Boys had significantly more impairment on the self-concept subscale than girls (44.7 ± 6.6 vs. 40.1 ± 9.9; t (63) = 2.12, *p* = 0.038). However, no significant differences were found between girls and boys in anxiety and depression symptoms. Regarding self-esteem, girls had a significantly lower self-esteem in the social subscale compared to boys (1.7 ± 0.93 vs. 1.1 ± 0.96; U = 247, *p* = 0.015). No significant differences were found between girls and boys in the general, parental, and academic self-esteem subscales or in the lie items and global self-esteem. No significant differences were found between girls and boys in cognitive or inattention problems. 

### 3.4. Sex by Age Interactions

When examining age correlations within each respective sex of interest, significant associations were found. For girls, functional inflexibility levels demonstrated a negative correlation with age (r = −0.548, *p* = 0.019) according to Pearson correlation analysis. Meanwhile, boys showed significant correlations between age and the following variables: age at tic onset (r = 0.362, *p* = 0.016), disruptive behaviors (r = −0.296, *p* = 0.046), and social self-esteem (r = −0.298, *p* = 0.044) using Spearman correlation analysis. However, no significant sex-by-age interactions were found for tic-related, internalizing, and externalizing symptom measures (all F’s < 3.1, all *p*-values > 0.08).

## 4. Discussion

The current study was conducted to evaluate sex-specific variations in tic onset and severity, psychological profiles, and quality of life among treatment-seeking youth diagnosed with persistent tic disorders. The results of this study add to the current body of research investigating the behavioral and psychopathological dimensions of tic disorders that manifest differently across sexes. The empirical evidence resulting from our analyses demonstrates that distinct sex-based patterns are evident in the clinical presentation of persistent tic disorders. Moreover, our study introduces new perspectives that potentially refine our understanding of sex differences in this population.

Our analyses highlighted notable trends in the presentation of tics across sexes. We observed that tic presentation does not significantly vary by sex in terms of onset age, motor and phonic tic severities, total tic severity, impairment rating, and global severity. This uniformity in tic presentation is supported by several recent studies of clinical youth populations [17,18,19,22,24]. Our results add to the existing body of scientific literature by reinforcing the notion that tics manifest similarly across sexes in the domains we investigated and the specific age range of the current study. This uniformity stands in contrast with other studies, which has often reported sex-specific differences in various aspects of tic disorders. Some report earlier tic onset [15,16], increased motor and phonic tic severity [17,18,19], and global tic severity in boys [18,19], while others have found motor, phonic, global tic severity, and tic-related impairment to be higher in girls [20,21]. It is important to acknowledge that our study focuses on a sample of youth actively seeking treatment, a group that may differ from the broader clinical populations examined in previous studies. This distinction highlights potential differences in the profiles and needs of treatment-seeking youth compared to the wider diagnosed population. Consequently, while our findings suggest a level of uniformity in tic presentation across sexes within this context, a consideration of the broader spectrum of tic disorder variability is warranted. Tics fluctuate over time and can be influenced by internal factors, such as psychological states, sensations, and thoughts, as well as external factors like environmental contexts and social interactions, which act as both triggers and modifiers of tic frequency and intensity [35]. They may also be influenced by genetics and cultural elements [1,36]. This dynamic nature highlights the importance of monitoring tic manifestations longitudinally to fully understand how their evolution and variability over time may differ across sexes.

Our analyses related to action planning styles, as assessed through the STOP questionnaire, revealed differences between boys and girls. Girls exhibited significantly higher levels of inflexibility and lower overall planning effectiveness compared to boys. However, no significant differences were found in over-preparedness, over-anticipation, and overactivity. This suggests that girls with tic disorders might experience distinct cognitive challenges that influence their daily planning and task management strategies. These differences may suggest that cognitive processing in girls and boys with tic disorders differ in a manner that significantly impacts their daily task management and problem-solving strategies. Furthermore, findings on quality of life demonstrate that the psychosocial burden of tics, particularly on psychological well-being and life satisfaction, is felt more acutely by girls. While it is well-established that children with tics experience a lower quality of life relative to their typically developing peers [14,37], evidence pertaining to sex differences in quality of life in TS is scarce. Multiple factors are crucial in understanding why and how tics can differentially impact quality of life in girls. Tics can affect concentration and performance [38], and this may disproportionally impact girls, given the high value placed on social relationships and acceptance during development [39,40]. 

Analyses pertaining to externalizing symptoms revealed a higher prevalence of explosive outbursts in boys. This contradicts previous studies showing no sex differences related to the prevalence of explosive outbursts [25,26]. This highlights the need for deeper exploration into the factors contributing to these behaviors, particularly within treatment-seeking samples. Furthermore, our study found no significant sex differences for anger and disruptive behaviors, which contrasts previous findings of increased disruptive behaviors in boys [19,23]. The latter two constructs were assessed with self-report inventories. Results may thus be influenced by poor insight. Additionally, we found that boys with tic disorders tend to exhibit more symptoms of hyperactivity while showing similar manifestations of oppositional behavior and the overall ADHD index compared to girls. This suggests that while boys may exhibit more ADHD symptoms, diagnostic rates do not necessarily differ between sexes. Because boys with tics may be more likely to also have more symptoms of hyperactivity, this could also explain why they may have more frequent explosive outbursts. These findings are consistent with the existing literature, which generally indicates a higher prevalence of ADHD symptoms among boys with tic disorders [17,18,19,20,23], and similar ADHD diagnoses in both sexes [27]. These results emphasize the complexity of diagnosing and understanding tic disorders, underscoring the importance of considering how societal expectations and norms may influence these processes.

Our analyses pertaining to internalizing symptoms revealed that boys had higher impairment in their self-concept compared to girls. Self-concept is a broad, comprehensive understanding that individuals construct about themselves, which is shaped over time by their experiences, the feedback they receive from others, and their social interactions [41]. Thus, boys with tic disorders may face greater challenges than girls in forming a positive view of themselves and in maintaining a strong sense of self-worth. Despite boys experiencing higher impairment in self-concept, girls reported having lower social self-esteem than boys. This implies that while girls may have had less difficulty with their overall self-concept, they may feel less confident or valued in social situations compared to boys. Prior research has shown that children with tic disorders and co-occurring conditions have poorer self-concept and self-esteem, regardless of the severity of their tics [41]. The presence of such co-occurring conditions, rather than the presence of tics, may thus account for difficulties with self-concept and self-esteem. This may also potentially explain sex differences on those latter measures. It is also possible that societal pressures to conform to masculinity ideals, alongside the discouragement of open discussions about mental health and emotional vulnerabilities, may contribute to the greater difficulties with self-concept in boys. Furthermore, our study showed that boys and girls with tic disorders had similar general, parental/at-home, academic, and global self-esteem. Additionally, our study found no significant differences between boys and girls with tic disorders in self-reported depression and anxiety symptoms, which contrasts with previous research indicating that girls with tic disorders exhibit more emotional problems, including higher incidences of anxiety and mood disorders [19,23,27]. Our results suggest that underlying factors affecting these aspects of well-being are complex and do not align directly with established expectations. This complexity highlights the importance of adopting a holistic approach in understanding and addressing the psychological impact of tic disorders, considering both sex-specific challenges and the broader societal context.

The analyses revealed significant age-related correlations within each sex, highlighting distinct patterns between girls and boys. For girls, the only notable finding was the negative correlation between age and functional inflexibility levels. This suggests that, over time, girls may exhibit increased rigidity or a decrease in adaptive flexibility. In contrast, boys demonstrated significant correlations between age and several variables. A positive correlation between age and the onset of tics in boys was found in our sample, indicating that older participants reported experiencing tic onset at an older age, while younger participants typically reported an earlier onset. This suggests a direct relationship between a participant’s current age and the age at which they first experienced tics. Additionally, disruptive behaviors and social self-esteem both exhibited negative correlations with age, suggesting improvements in these areas as boys age. These differences may reflect the natural maturation process or the effectiveness of targeted interventions over time.

Lastly, we found that tic-related measures did not significantly differ by sex or age, indicating a consistent presentation of tic disorders in childhood and early adolescence for both boys and girls. This contrasts previous findings showing a greater increase in tic severity with age in girls relative to boys [20]. It is possible that such differentiation across sexes occurs later in adolescence and would thus not be captured in the current study. We also did not find age-by-sex interactions related to external and internal symptom measures. These findings suggest a reevaluation of how developmental stages and sex differences influence tic disorders, requiring further research into the complexities of these conditions across various demographic groups and examines the multifaceted factors that shape symptom presentations over time.

### 4.1. Strengths and Limitations

Our study involved a comprehensive exploration of the cognitive and psychosocial aspects of tic disorders while incorporating underutilized but highly informative questionnaires in the context of sex differences. Nonetheless, our study has some limitations. First, the generalizability of our findings is limited by the sample size and the fact that our study has an uneven sex distribution, with fewer girls (N = 19) compared to boys (N = 47). This disparity does reflect the prevalence of tic disorders, which are generally more common in males. However, the underrepresentation of girls in our sample may restrict the applicability of our findings. Second, our study sample comprises treatment-seeking youth, which may introduce a recruitment bias. Our findings may not be representative of all youths with tics, given that treatment-seeking individuals might exhibit enhanced tic severity or psychosocial difficulties. Third, given the broad score of our analyses and the relatively small sample sizes, we did not apply correction for multiple comparisons.

### 4.2. Future Directions

Given these limitations, we invite replication of the current results in larger and diverse samples of youths with tics to ensure that findings are representative of the broader tic population. Incorporating neuropsychological measurements and assessments of emotional regulation could deepen our understanding of the mechanisms underlying tic expression and its variations. Additionally, exploring the potential impact of hormonal fluctuations on tics may shed light on the biological underpinnings of sex differences. We also recommend that future studies investigate the interactions of medication with sex differences, as this could provide valuable insights into tailored treatment approaches. Longitudinal studies, including those that examine cultural influences on gender roles, would be important to elucidate the progression of tics and developmental trends across sexes. Such studies could provide a clearer prognosis for boys and girls diagnosed with tic disorders.

## 5. Conclusions

Our findings further demonstrate the complex nature of tic disorders, characterized by a combination of shared and distinct experiences across sexes. These nuanced differences and similarities necessitate a multidisciplinary approach to research, diagnosis, and treatment within this population. By deepening our understanding of these multifaceted disorders and highlighting the differences in their manifestations, we can enhance the detection of tic disorders. This would help address the specific needs of youth and improve interventions. Ultimately, such targeted strategies aim to improve their functioning, quality of life, and psychological well-being. 

## Figures and Tables

**Table 1:** Sex differences in tic-related measures.

	All N = 66	Girls N = 19	Boys N = 47	Test Statistic	*p*-Value
**Preliminary interview**					
Age of tic onset	5.7 (2.1)	5.4 (2.3)	5.8 (2.0)	379 ^a^	0.551
**YGTSS**					
*Clinical interview with youth*					
Motor tic severity	11.3 (4.1)	11.8 (5.6)	11.2 (3.4)	0.539 ^b^	0.592
Phonic tic severity	7.8 (5.3)	7.2 (5.7)	8.0 (5.2)	412 ^a^	0.618
Total tic severity	19.1 (7.8)	19.0 (9.5)	19.1 (7.1)	−0.082 ^b^	0.935
Impairment	12.5 (11.7)	13.7 (10.5)	12.0 (12.2)	385 ^a^	0.432
Global severity	31.5 (15.8)	32.7 (17.7)	31.1 (15.1)	420 ^a^	0.806
*Clinical interview with parents*					
Motor tic severity	11.6 (3.8)	11.6 (4.2)	11.7 (3.8)	−0.115 ^b^	0.909
Phonic tic severity	8.3 (5.7)	7.7 (5.8)	8.6 (5.6)	414 ^a^	0.734
Total tic severity	20.0 (7.5)	19.2 (7.5)	20.3 (7.6)	−0.503 ^b^	0.617
Impairment	16.6 (11.2)	17.2 (9.7)	16.3 (11.8)	386 ^a^	0.670
Global severity	36.1 (14.1)	36.4 (14.1)	36.0 (14.3)	0.097 ^b^	0.923
**STOP**					
Over preparedness	29.0 (7.4)	27.3 (7.5)	29.7 (7.3)	−1.13 ^b^	0.261
Inflexibility	42.9 (7.5)	39.8 (8.3)	44.1 (6.2)	−2.12 ^b^	0.038 *
Over anticipation	24.8 (5.7)	24.1 (5.0)	25.1 (6.0)	−0.631 ^b^	0.530
Over activity	7.2 (12.1)	4.9 (3.2)	8.1 (14.1)	303 ^a^	0.093
Global score	111 (16.9)	102 (14.3)	114 (16.9)	255 ^a^	0.017 *
**GTS-QoL**					
Psychological	30.2 (17.3)	39.4 (20.4)	26.6 (14.6)	252 ^a^	0.015 *
Physical	22.3 (15.0)	26.8 (14.1)	20.6 (15.1)	290 ^a^	0.062
Obsessive-compulsive	24.9 (16.4)	24.2 (14.3)	25.2 (17.4)	412 ^a^	0.970
Cognitive	23.3 (17.0)	27.8 (18.5)	21.6 (16.3)	320 ^a^	0.157
Life satisfaction	26.2 (13.2)	31.6 (14.7)	24.0 (12.1)	2.12 ^b^	0.038 *

^a^ Mann–Whitney U test; ^b^ *t*-test; * *p*-value < 0.05. Abbreviations: YGTSS, Yale Global Tic Severity Scale, STOP, Style of Planning Questionnaire; GTS-QoL, Gilles de la Tourette Syndrome-Quality of Life Scale.

**Table 2:** Sex differences in externalizing symptoms.

	All N = 66	Girls N = 19	Boys N = 47	Test Statistic	*p*-Value
**Preliminary interview**					
Explosive outbursts	31 (47.7%)	5 (26.3%)	26 (56.5%)	4.92 ^c^	0.027 *
**Beck Youth Inventories**					
Anger	12.4 (8.5)	13.7 (9.3)	11.9 (8.3)	391 ^a^	0.506
Disruptive behaviours	4.7 (4.3)	4.1 (3.9)	4.9 (4.5)	386 ^a^	0.460
**CONNERS**					
Oppositional	7.9 (4.6)	6.3 (4.5)	8.5 (4.6)	278 ^a^	0.080
Hyperactivity	5.3 (3.9)	3.7 (3.2)	5.8 (3.9)	252 ^a^	0.030 *
Index DAH	15.7 (8.6)	14.1 (7.6)	16.3 (8.9)	−0.872 ^b^	0.387

Note: Data are N (%) for explosive outbursts. ^a^ Mann–Whitney U test; ^b^ *t*-test; ^c^ chi-square test; * *p*-value < 0.05.

**Table 3:** Sex differences in internalizing symptoms.

	All N = 66	Girls N = 19	Boys N = 47	Test Statistic	*p*-Value
**Beck Youth Inventories**					
Self-concept	43.4 (7.9)	40.1 (9.9)	44.7 (6.6)	−2.12 ^b^	0.038 *
Anxiety	15.7 (8.5)	17.7 (9.0)	14.9 (8.2)	329 ^a^	0.117
Depression	11.0 (8.0)	13.8 (10.1)	9.8 (6.7)	319 ^a^	0.087
**CFSEI-II**					
General self-esteem	1.8 (2.0)	2.5 (3.3)	1.5 (1.8)	294 ^a^	0.118
Parental self-esteem	0.41 (0.71)	0.59 (0.87)	0.35 (0.64)	335 ^a^	0.284
Academic self-esteem	0.62 (0.81)	0.59 (0.87)	0.63 (0.80)	370 ^a^	0.709
Social self-esteem	1.2 (0.98)	1.7 (0.93)	1.1 (0.96)	247 ^a^	0.015 *
Lie items	1.2 (1.3)	0.94 (1.2)	1.3 (1.3)	330 ^a^	0.318
Global self-esteem	4.0 (3.7)	5.3 (4.6)	3.5 (3.3)	298 ^a^	0.144
**CONNERS**					
Cognitive/inattention problems	7.5 (4.9)	6.9 (5.0)	7.8 (5.0)	354 ^a^	0.566

Note: Data are N (%) for explosive outbursts. ^a^ Mann–Whitney U test; ^b^ *t*-test; * *p*-value < 0.05. Abbreviation: CFSEI-II, Culture-Free Self-Esteem Inventories, 2nd edition.

## Data Availability

The data presented in this study are available on request from the corresponding author, upon reasonable request.
